# Anti-HER2 CD4^+^ T-helper type 1 response is a novel immune correlate to pathologic response following neoadjuvant therapy in HER2-positive breast cancer

**DOI:** 10.1186/s13058-015-0584-1

**Published:** 2015-05-23

**Authors:** Jashodeep Datta, Erik Berk, Shuwen Xu, Elizabeth Fitzpatrick, Cinthia Rosemblit, Lea Lowenfeld, Noah Goodman, David A Lewis, Paul J Zhang, Carla Fisher, Robert E Roses, Angela DeMichele, Brian J Czerniecki

**Affiliations:** Department of Surgery, University of Pennsylvania Perelman School of Medicine, Rena Rowen Breast Center, 3400 Civic Center Drive, Philadelphia, PA 19104 USA; Division of Hematology/Oncology, Department of Medicine, University of Pennsylvania Perelman School of Medicine, Philadelphia, PA USA; Department of Epidemiology and Biostatistics, University of Pennsylvania Perelman School of Medicine, Philadelphia, PA USA; Department of Pathology, University of Pennsylvania Perelman School of Medicine, Philadelphia, PA USA; Rena Rowen Breast Center, Hospital of the University of Pennsylvania, Philadelphia, PA USA

## Abstract

**Introduction:**

A progressive loss of circulating anti-human epidermal growth factor receptor-2/*neu* (HER2) CD4^+^ T-helper type 1 (Th1) immune responses is observed in HER2^pos^-invasive breast cancer (IBC) patients relative to healthy controls. Pathologic complete response (pCR) following neoadjuvant trastuzumab and chemotherapy (T + C) is associated with decreased recurrence and improved prognosis. We examined differences in anti-HER2 Th1 responses between pCR and non-pCR patients to identify modifiable immune correlates to pathologic response following neoadjuvant T + C.

**Methods:**

Anti-HER2 Th1 responses in 87 HER2^pos^-IBC patients were examined using peripheral blood mononuclear cells pulsed with 6 HER2-derived class II peptides via IFN-γ ELISPOT. Th1 response metrics were anti-HER2 *responsivity*, *repertoire* (number of reactive peptides), and *cumulative response* across 6 peptides (spot-forming cells [SFC]/10^6^ cells). Anti-HER2 Th1 responses of non-pCR patients (n = 4) receiving adjuvant HER2-pulsed type 1-polarized dendritic cell (DC1) vaccination were analyzed pre- and post-immunization.

**Results:**

Depressed anti-HER2 Th1 responses observed in treatment-naïve HER2^pos^-IBC patients (n = 22) did not improve globally in T + C-treated HER2^pos^-IBC patients (n = 65). Compared with adjuvant T + C receipt, neoadjuvant T + C — utilized in 61.5 % — was associated with higher anti-HER2 Th1 repertoire (p = 0.048). While pCR (n = 16) and non-pCR (n = 24) patients did not differ substantially in demographic/clinical characteristics, pCR patients demonstrated dramatically higher anti-HER2 Th1 responsivity (94 % vs. 33 %, p = 0.0002), repertoire (3.3 vs. 0.3 peptides, p < 0.0001), and cumulative response (148.2 vs. 22.4 SFC/10^6^, p < 0.0001) versus non-pCR patients. After controlling for potential confounders, anti-HER2 Th1 responsivity remained independently associated with pathologic response (odds ratio 8.82, p = 0.016). This IFN-γ^+^ immune disparity was mediated by anti-HER2 CD4^+^T-bet^+^IFN-γ^+^ (i.e., Th1) — not CD4^+^GATA-3^+^IFN-γ^+^ (i.e., Th2) — phenotypes, and not attributable to non-pCR patients’ immune incompetence, host-level T-cell anergy, or increased immunosuppressive populations. In recruited non-pCR patients, anti-HER2 Th1 repertoire (3.7 vs. 0.5, p = 0.014) and cumulative response (192.3 vs. 33.9 SFC/10^6^, p = 0.014) improved significantly following HER2-pulsed DC1 vaccination.

**Conclusions:**

Anti-HER2 CD4^+^ Th1 response is a novel immune correlate to pathologic response following neoadjuvant T + C. In non-pCR patients, depressed Th1 responses are not immunologically “fixed” and can be restored with HER2-directed Th1 immune interventions. In such high-risk patients, combining HER2-targeted therapies with strategies to boost anti-HER2 Th1 immunity may improve outcomes and mitigate recurrence.

**Electronic supplementary material:**

The online version of this article (doi:10.1186/s13058-015-0584-1) contains supplementary material, which is available to authorized users.

## Introduction

Human epidermal growth factor receptor-2 (HER2)/*neu* overexpression, a molecular oncodriver in 20–25 % of breast cancers (BC) [1], is associated with an aggressive clinical course and poor overall prognosis [2]. The availability of HER2-targeted therapies (e.g., trastuzumab, lapatinib, etc.) has dramatically improved outcomes in patients with HER2-positive (HER2^pos^) BC [3, 4]. In contemporary practice, patients with larger resectable tumors often benefit from neoadjuvant administration of trastuzumab and chemotherapy (T + C), with nearly 40–60 % achieving pathologic complete response (pCR) [5–7]; compared with incomplete response (non-pCR), pCR is associated with decreased recurrence and improved long-term survival [7, 8]. While absent estrogen/progesterone receptor (ER/PR) expression appears to reproducibly correlate with pCR [8, 9], there are a paucity of modifiable immune signatures that are associated with response and/or resistance to neoadjuvant T + C.

Utilizing a prospective cohort, we have recently demonstrated a progressive loss in anti-HER2 CD4^+^ T-helper type-1 (Th1) immunity across a tumorigenesis continuum in HER2^pos^ BC [10]. Interestingly, HER2-specific Th1 responses are preserved in healthy volunteers and patients harboring HER2^neg^ (0–1+) invasive breast cancer (IBC). In patients with HER2^pos^ IBC, this anti-HER2 Th1 deficit is not impacted by standard therapies (i.e., surgical resection, radiation, or T + C treatment), but can be restored following HER2-pulsed type-1-polarized dendritic cell (DC1) vaccinations. Moreover, depressed anti-HER2 Th1 responses predict an increased risk of subsequent recurrence in patients treated with adjuvant T + C [10]. These observations prompted us to investigate whether similar depressed anti-HER2 Th1 responses are observed in another known harbinger of recurrence, non-pCR status following neoadjuvant T + C [8]; conversely, we hypothesized that preservation/restoration of anti-HER2 Th1 responses may be associated with pCR.

In this study, we identified elevated anti-HER2 CD4^+^ Th1 response as a novel systemic immune correlate to pCR following neoadjuvant T + C in patients with HER2^pos^ IBC. Relatively depressed anti-HER2 Th1 responses in patients with non-pCR are not attributable to host-level T cell anergy, loss of immunocompetence, or increase in circulating immunosuppressive phenotypes. Importantly, this anti-HER2 Th1 deficit in patients with non-pCR is not fixed, and can be corrected with CD4^+^ Th1-directed immune manipulations via HER2-targeted DC1 vaccinations. To the best of our knowledge, these observations represent the first demonstration of a modifiable host-level oncodriver (HER2/*neu*)-specific immune disparity that is associated with pathologic response to neoadjuvant T + C. These findings may have important implications for immune monitoring and/or design of adjunctive immune therapies to improve outcomes in trastuzumab-treated HER2^pos^ BC patients.

## Methods

### Study design

**Fig. 1:**
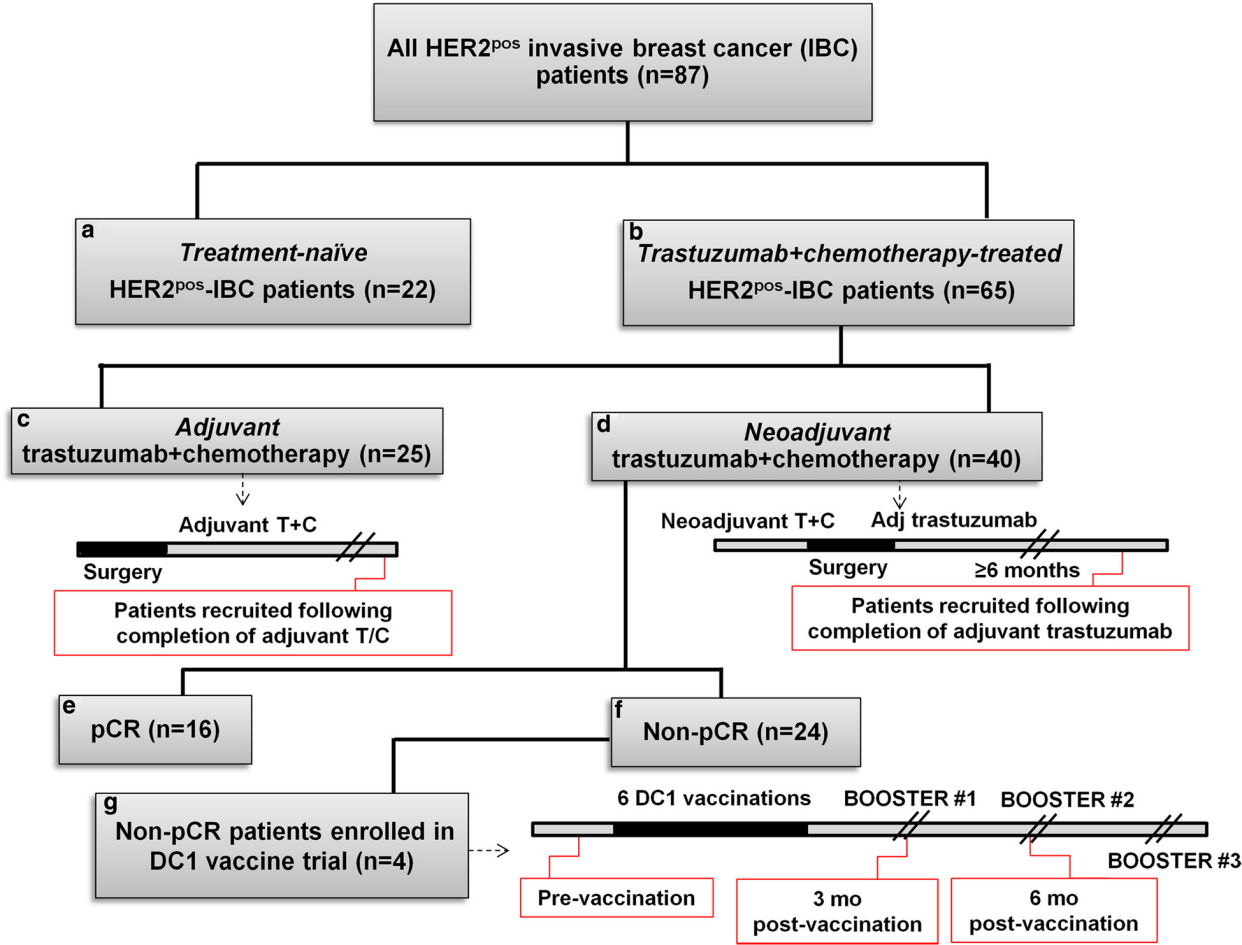
*Consolidated Standards of Reporting Trials* (CONSORT) diagram of the study population. In this study, 87 patients with human epidermal growth factor receptor 2-positive (*HER2* ^*pos*^) breast cancer were enrolled; all tumors were histologically confirmed as invasive breast cancer (*IBC*) with HER2 overexpression (3+ or 2+/fluorescence in situ hybridization (FISH)-positive). Cohorts are labeled (**a**–**g**) for ease of comparison (of immune responses), and are referred to in *Results*. Time points at which blood was drawn are indicated (*red callout boxes*). Median follow up in the cohort treated with trastuzumab and chemotherapy (*T + C*) was 26 (IQR 16.5–31.0) months. *pCR* pathologic complete response, *DCI* type 1-polarized dendritic cell, *Adj* adjuvant, *mo* months

After approval by the Institutional Review Board of the University of Pennsylvania, 87 patients with HER2^pos^ IBC were enrolled in a non-biased fashion (Table 1). Eligible patients had histologically confirmed IBC, HER2/*neu* overexpression (i.e., immunochemistry (IHC) 3+ or 2+/fluorescence in situ hybridization (FISH)-positive) confirmed at our institution, no evidence of distant metastasis, and were not receiving immunosuppressive medications. Informed consent was obtained from all participants. Anti-HER2 CD4^+^ Th1 responses of recruited subjects were analyzed prospectively. Anti-HER2 Th1 responses in *treatment-naïve* (i.e., not receiving definitive therapy at enrollment) stage I–III HER2^pos^ IBC patients (n = 22) were established as an immunologic "baseline", and were compared with Th1 responses in stage I–III HER2^pos^ IBC patients who had *completed T + C treatment* (n = 65; i.e., either neoadjuvant or adjuvant T + C plus definitive surgery). In patients treated with T + C, analyses were stratified by sequence of chemotherapy (i.e., neoadjuvant versus adjuvant), and further sub-stratified by pCR and non-pCR status within the neoadjuvant cohort (Fig. 1). pCR was defined as absence of residual invasive cancer on pathologic examination of resected breast specimen(s) and sampled lymph nodes (i.e., ypT0/Tis ypN0).

**Table 1:** Demographic and tumor-related characteristics of the study population: age, race, AJCC pathologic stage, hormone receptor status, and time from completion of trastuzumab (when applicable)

Characteristic	Treatment-naïve HER2^pos^ IBC (n = 22)	HER2^pos^ IBC treated with T + C (n = 65)	
		Neoadjuvant (n = 40)	Adjuvant (n = 25)
Age, years, mean ± standard error	56.8 ± 3.1	45.9 ± 2.1	57.2 ± 2.6
Age, years, range	36–88	24–81	28–85
Race/ethnicity, number (%)			
Caucasian	17 (77.3)	35 (87.5)	19 (76.0)
African-American	2 (9.1)	3 (7.5)	4 (16.0)
Asian	2 (9.1)	1 (2.5)	0 (0)
Hispanic	1 (4.5)	1 (2.5)	2 (8.0)
AJCC stage at diagnosis^a^, number (%)			
Stage 1	14 (63.6)	0 (0)	6 (24.0)
Stage 2	6 (27.3)	21 (52.5)	15 (60.0)
Stage 3	2 (9.1)	19 (47.5)	4 (16.0)
Hormone receptor status, number (%)			
ER/PR^pos^	12 (54.5)	18 (45.0)	11 (44.0)
ER/PR^neg^	10 (45.5)	22 (55.0)	14 (56.0)
Time from completion of trastuzumab to study enrollment, number (%)			
<6 months		18 (45.0)	10 (40.0)
≥6 months		22 (55.0)	15 (60.0)

^a^For neoadjuvant cohort, AJCC clinical stage is shown. *HER2* ^*pos*^ Human epidermal growth factor receptor overexpressing, *IBC* invasive breast cancer, *T + C* trastuzumab and chemotherapy, *AJCC* American Joint Committee on Cancer, *ER* estrogen receptor, *PR* progesterone receptor

Four patients with non-pCR were recruited to our adjuvant HER2-pulsed DC1 vaccination trial (NCT02061423); anti-HER2 Th1 responses in these patients were compared pre-immunization and post-immunization.

### Immune response detection

Circulating anti-HER2 CD4^+^ Th1 responses were examined in unexpanded peripheral blood mononuclear cells (PBMC) pulsed ex vivo with six HER2-derived class II peptides (42–56, 98–114, 328–345, 776–790, 927–941, 1166–1180 [11]), by measuring IFN-γ production via enzyme-linked immunosorbent spot (ELISPOT) assays. ELISPOT was performed as previously described [10, 12]. Briefly, polyvinylidene fluoride (PVDF) membrane plates (Mabtech Inc., Cincinnati, OH, USA) were coated with anti-IFN-γ capture antibody. After plates were blocked, cryopreserved PBMCs, isolated using density gradient centrifugation, were plated in triplicate (2 × 10^5^ cells/well) and incubated at 37 °C for 24–36 h with either HER2 peptides (4 μg; Genscript, Piscataway, NJ, USA); media alone (unstimulated control); or positive control (anti-human CD3/CD28 antibodies (0.5 μg/mL; BD Pharmingen, San Jose, CA, USA)). After washing, biotinylated detection antibody (100 μg/mL) and 1:1000-diluted streptavidin-horseradish peroxidase (HRP) in PBS + 0.5 % FCS were added serially, and addition of 3,3′-5,5′ tetramethylbenzidin (TMB) substrate solution revealed spot formation. Spot-forming cells (SFC) were counted using an automated reader (ImmunoSpot CTL, Shaker Heights, OH, USA).

PBMC from HLA-A2.1^pos^ donors were stimulated with two HER2-derived class I peptides (369–377, 689–697) [11]; phorbol-12-myristate 13-acetate (PMA, 50ng/mL) and ionomycin (1μg/mL; Sigma-Aldrich, St. Louis, MO, USA) served as positive control. HLA-A2.1 typing (LABType® SSO) was performed in the Clinical Immunology laboratory at the Hospital of the University of Pennsylvania. In addition, HER2-specific IL-4 and IL-10 production (surrogates for T-helper type-2 (Th2) and regulatory T-cell (T_reg_) cell function, respectively) were measured by ELISPOT [13]. Recall Th1 responses were examined by stimulating PBMC with 1:100-diluted recall stimuli *Candida albicans* (Allermed Laboratories, San Diego, CA, USA) and tetanus toxoid (Santa Cruz Biotechnology, Dallas, TX, USA).

An empiric method of determining anti-HER2 Th1 response specificity was employed [10]. A positive response to an individual HER2 peptide was defined as: (a) threshold minimum of 20 SFC/2 × 10^5^ cells in experimental wells after subtracting unstimulated background; and (b) ≥2-fold increase in antigen-specific SFCs over background. Three metrics of anti-HER2 Th1 response were defined for each cohort: (a) responsivity (proportion of patients responding to ≥1 peptide), (b) repertoire (mean number of reactive peptides), and (c) cumulative response across 6 peptides (SFC/10^6^ cells). A sample calculation is illustrated in Additional file 1: Figure S1. Inter-assay precision of ELISPOT assays was validated as described previously [14].

### Flow cytometry

PBMC suspensions were prepared in FACS buffer (PBS + 1 % FCS + 0.01 % azide) and anti-human CD3, CD4, CD8, CD83, HLA-DR, CD11b, CD33, CD19, CD16 (BD Bioscience, San Jose, CA, USA), CD4, and CD25 (Biolegend, San Diego, CA, USA) were used to determine the relative PBMC immunophenotype. After washing, cells were incubated for 30 minutes at room temperature (RT) with antibody mixtures. Following incubation, cells were washed/fixed with 2 % paraformaldehyde. Intracellular staining with anti-FoxP3 (eBioscience, San Diego, CA, USA) using the FoxP3 fixation/permeabilization kit (Biolegend) was performed according to manufacturer’s instructions. Analysis was performed using the BD LSR-II cytometer, and datasets were analyzed using CellQuest Pro software.

### Th1 vs Th2 contributions to anti-HER2 IFN-γ^+^ T cells

PBMC were resuspended at 1.2 × 10^6^ cells/mL in DMEM + 5 % human serum in 24-well plates, and pulsed with HER2-class II peptide mix (24 μg/mL). Unstimulated and anti-CD3/CD28 antibody-pulsed PBMCs from each donor served as negative and positive controls, respectively. Following incubation for 6 h at 37 °C, protein transport inhibitor Brefeldin-A (Sigma Aldrich; 10 μg/mL) was added to each sample, and incubated overnight. Following washing, cells were stained with anti-CD4 for 30 minutes at RT. Cells were washed twice, fixed and permeabilized as described above, and stained with anti-T-bet, anti-GATA-3 and anti-IFN-γ (Biolegend) for 30 minutes. After incubation, cells were washed and analyzed using the BD LSR-II cytometer.

### Vaccination procedure and trial design

We have initiated a phase I adjuvant HER2-pulsed DC1 vaccination trial for patients with HER2^pos^ IBC with residual disease following neoadjuvant T + C (NCT02061423). Eligible patients are 18 years or older, have Eastern Cooperative Oncology Group (ECOG) performance status score of 0 or 1, and have biopsy-proven stage I–III HER2^pos^ IBC. The primary endpoint of this trial is safety/feasibility; however, we report an interim analysis of anti-HER2 immune responses following vaccination (a secondary endpoint) in recruited patients (n = 4) as proof of principle of its immunogenicity in this heavily pre-treated population.

Monocytic dendritic cell precursors (CD14^pos^ peripheral blood monocytes) were obtained from subjects via tandem leukapheresis/countercurrent centrifugal elutriation. Dendritic cells (DCs) were cultured overnight in macrophage serum-free medium (Cellgro, Manassas, VA, USA) with granulocyte monocyte colony stimulating factor (GM-CSF, 250 IU/mL; Berlex, San Pablo, CA, USA) and IL-4 (1000 u/mL; R&D Systems, Minneapolis, MN, USA) - these are considered immature DCs (iDCs). The following day, iDCs were pulsed with the aforementioned six HER2 major histocompatability class (MHC)-II promiscuous-binding peptides (42–56, 98–114, 328–345, 776–790, 927–941, 1166–1180). After 8–12 h incubation, IFN-γ (1000 U/mL) was added; the following day, National Institutes of Health (NIH) reference standard lipopolysaccharide (LPS) was added (10 ng/mL) to achieve full DC activation to a DC1 phenotype 6 h before harvest. For HLA-A2.1^pos^ patients, DC1 were pulsed with two MHC class I binding peptides (369–377, 689–697). Harvested cells were washed and lot release criteria of >70 % viability, negative Gram stain, and endotoxin <5 EU/kg confirmed.

Immunizations were administered in the NIH-designated General Clinical Research Center at the Hospital of the University of Pennsylvania. Injections comprised 10–20 × 10^6^ HER2-pulsed DC1s suspended in 1 mL sterile saline, and administered by ultrasound guidance into groin lymph nodes [12, 15]. Immunizations were administered once weekly for 6 weeks, followed by three booster doses spaced 3 months apart.

### Statistical analysis

Descriptive statistics summarized distributions of patient characteristics and immune response variables. Data transformation of the cumulative response variable (natural log or square root) was applied to meet the assumptions of parametric testing, where applicable. The unpaired or paired Student’s *t* test (parametric continuous data), Mann–Whitney (non-parametric continuous data), and chi square (*χ*^2^) tests (categorical data) were used for two-group and univariate comparisons between pCR and non-pCR cohorts. To determine independent correlates of pCR, variables with a trend toward significance on univariate testing (*p* <0.20) were entered into a forward, stepwise multivariable logistic regression model (*p* <0.05 for entry, *p* <0.10 for exit). A *p* value <0.05 was considered statistically significant. All tests were two-sided. Analyses were performed using Prism 5.0 (GraphPad Inc., La Jolla, CA, USA) and SPSS version 22 (IBM Corp, Chicago, IL, USA).

## Results

### Patient characteristics

In the overall cohort (n = 87), mean age was 51.4 ± 1.5 (range 24–88) years and a majority (81.6 %) were white. Demographic and tumor-related characteristics of participants are detailed in Table 1. Of the cohort treated with T + C (n = 65), neoadjuvant T + C was administered in 40 patients (61.5 %); 16 patients (40.0 %) achieved pCR whereas 24 (60.0 %) had residual disease at surgery (non-pCR). Median follow up in patients treated with T + C was 26 (IQR 16.5–31) months (Fig. 1).

In patients treated with neoadjuvant T + C, mean age and body mass index (BMI) were 45.9 ± 2.1 years and 32.3 ± 2.1 kg/m^2^, and a majority were white (87.5 %) or premenopausal (57.5 %). More than half of patients had ER/PR^neg^ tumors (55.0 %) or clinical stage II disease at diagnosis (52.5 %). Among pathologic features, lymphovascular invasion (LVI) and nuclear grade 3 were observed in 7 (17.5 %) and 26 (65.0 %) tumors, respectively. The most commonly utilized treatment regimen and operative approach was Adriamycin/Cyclophosphamide/Taxol/Herceptin (AC/TH; 82.5 %) and mastectomy (67.5 %), respectively.

### Depressed anti-HER2 CD4^+^ responses in treatment-naïve HER2^pos^ IBC patients are not globally restored following T + C

**Fig. 2:**
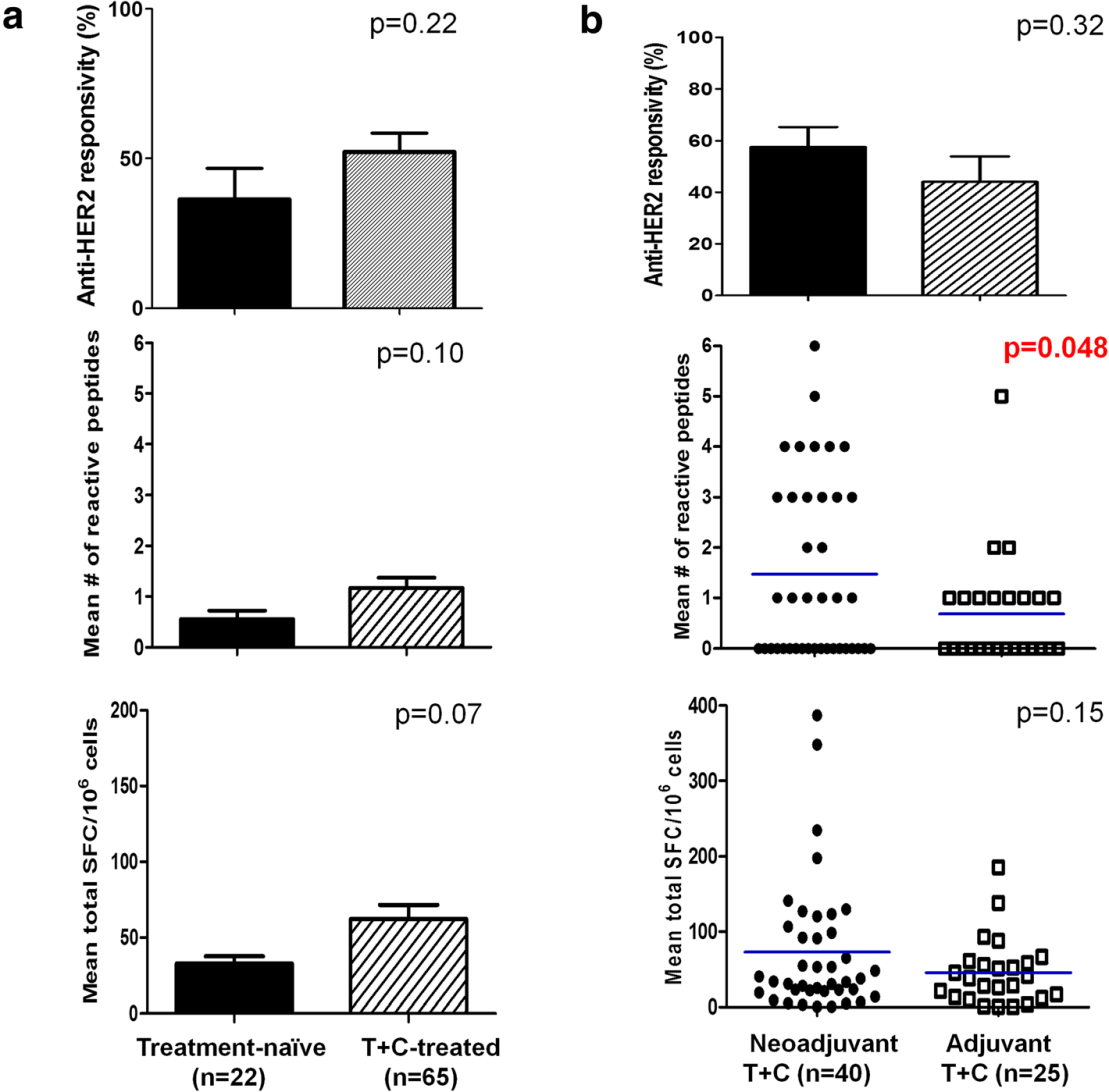
Interferon (IFN)-γ^+^ anti-human epidermal growth factor receptor 2 (*HER2*) CD4^+^ T cell response variations in HER2^pos^ patients with invasive breast cancer (IBC). IFN-γ enzyme-linked immunosorbent spot analysis of HER2 peptide-pulsed peripheral blood mononuclear cells examined anti-HER2 CD4^+^ T cell responses in patients with HER2^pos^ IBC, stratified by *anti-HER2 responsivity*, response repertoire (*mean # of reactive peptides*), and cumulative response (*mean total SFC/10* ^*6*^ *cells*). Differences between (**a**) *Treatment-naïve* patients with HER2^pos^ IBC (n = 22) and those treated with trastuzumab and chemotherapy (*T + C*) (n = 65); and (**b**) patients treated with *Neoadjuvant T + C* (n = 40) and those treated with *Adjuvant T + C* (n = 25). *SFC* spot-forming cells

Using PBMCs, we compared IFN-γ^+^ anti-HER2 CD4^+^ T-cell responses between HER2^pos^ IBC cohorts via HER2-stimulated IFN-γ ELISPOT. We have previously demonstrated a striking loss of anti-HER2 Th1 responses in treatment-naïve HER2^pos^ IBC patients relative to healthy donors [10]. In the current study, depressed anti-HER2 Th1 responses in treatment-naïve HER2^pos^ IBC patients (*cohort A*; Fig. 1) – assessed by responsivity, repertoire, or cumulative response – did not improve globally in HER2^pos^ IBC patients treated with T + C (*cohort B*; Fig. 2a). Among T + C – treated patients, neoadjuvant T + C receipt (*cohort D*) was associated with higher anti-HER2 Th1 repertoire (1.5 ± 0.3 vs. 0.8 ± 0.4; *p* = 0.048), but not responsivity or cumulative response, compared with adjuvant T + C treatment (*cohort C*; Fig. 2b).

### Anti-HER2 T cell immune responses correlate strongly with pCR

**Fig. 3:**
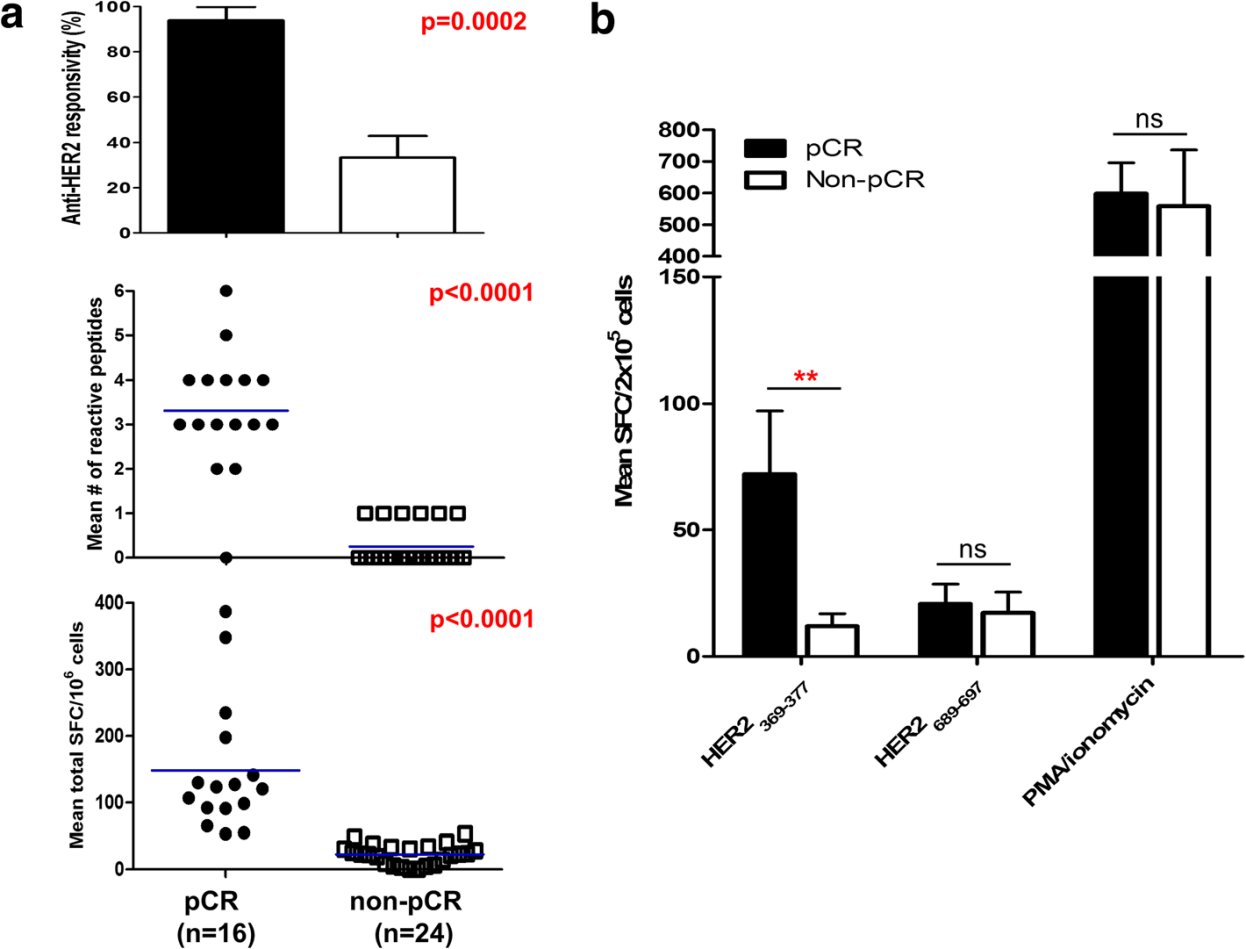
Significant disparity in anti-human epidermal growth factor receptor 2 (*anti-HER*2) interferon (IFN)-γ^+^ T cell immune responses between patients with pathologic complete response (*pCR*) and patients with *non-pCR*. **a** Significantly elevated anti-HER2 CD4^+^ T cell responses by IFN-γ enzyme-linked immunosorbent spot analysis (ELISPOT) are observed in patients with HER2^pos^ invasive breast cancer achieving pCR (n = 16) following neoadjuvant trastuzumab and chemotherapy (T + C), compared with patients with non-pCR (n = 24). Peripheral blood mononuclear cells (PBMC) from patients with pCR and non-pCR were stimulated ex vivo with six HER2-derived class II peptides and IFN-γ production via ELISPOT was compared. Responses are stratified by anti-HER2 responsivity, repertoire, and cumulative response. **b** Evaluable HLA-A2.1^pos^ PBMC from patients with pCR (n = 6; *black bars*) and non-pCR (n = 4; *white bars*) were stimulated ex vivo with two HER2-derived class I peptides, *HER2*_*369*–*377*_ and *HER2*_*689–697*,_ and IFN-γ production via ELISPOT was compared. Phorbol-12-myristate 13-acetate (*PMA*) and ionomycin served as positive control. Results are expressed as mean spot-forming cells (*SFC*)/2 × 10^5^ cells ± standard error of the mean

In the cohort receiving neoadjuvant T + C, patients achieving pCR (*cohort E*; Fig. 1) demonstrated dramatically higher IFN-γ^+^ anti-HER2 Th1 responsivity (93.8 % vs 33.3 %, *p* = 0.0002), repertoire (3.3 ± 0.3 vs 0.3 ± 0.1, *p* <0.0001), and cumulative response (148.2 ± 24.6 vs 22.4 ± 3.0, *p* <0.0001) compared with non-pCR patients (*cohort F*; Fig. 3a). Of note, median duration from initiation of neoadjuvant T + C to study enrollment did not differ between pCR and non-pCR cohorts (23.5 vs 26.5 months, *p* = 0.44).

Evaluable PBMC from HLA-A2.1^pos^ pCR and non-pCR patients were stimulated ex vivo with two HER2-derived class I peptides. Compared with non-pCR patients, IFN-γ^+^ CD8^+^ T cell responses were significantly higher in pCR patients upon stimulation with the immunodominant HER2_369–377_ (72.1 ± 10.2 vs 12.1 ± 2.4 SFC/2 × 10^5^, *p* = 0.002) epitope, but not the subdominant HER2_689–697_ (21.5 ± 3.7 vs 17.3 ± 4.0 SFC/2 × 10^5^, *p* = 0.47) epitope (Fig. 3b).

### Anti-HER2 Th1 responsivity is independently associated with pCR following multivariable analysis

The independent association between IFN-γ^+^ anti-HER2 Th1 responses and pCR was evaluated by controlling for confounding from relevant demographic and clinicopathologic characteristics. Upon univariate testing, pCR and non-pCR cohorts did not differ significantly by age, menopausal status, race, BMI, comorbidity, presence of LVI, nuclear grade, or utilized T + C regimens. However, pCR patients were more likely to have ER/PR^neg^ tumors compared with patients with non-pCR (68.8 % vs 29.2 %, *p* = 0.02). Although pCR patients demonstrated a trend toward presentation at lower (i.e., stage II) clinical stage (68.8 % vs 41.7 %, *p* = 0.12) and less frequent need for mastectomy (50.0 % vs 79.2 %, *p* = 0.09), these comparisons did not reach statistical significance (Table 2).

**Table 2:** Univariate comparison of demographic and tumor-related characteristics between pCR and non-pCR patients

Characteristic	Patients with pCR, number (%) or median (IQR)	Patients with non-pCR, number (%) or median (IQR)	*P* value
Overall population	16 (40)	24 (60)	--
Age (years), median	45.5 (35.5–51.5)	49.5 (34.3–53.0)	0.97
BMI (kg/m^2^), median	25.7 (23.3–29.6)	27.3 (24.2–31.0)	0.49
Race			0.63
White	15 (93.8)	20 (83.3)	
Black/Asian/Hispanic	1 (6.2)	4 (16.7)	
Charlson comorbidity index			0.75
<2 (mild)	7 (43.8)	9 (37.5)	
≥2 (moderate–high)	9 (56.2)	15 (62.5)	
Menopausal status			1.00
Premenopausal	9 (56.2)	14 (58.3)	
Postmenopausal	7 (43.8)	10 (41.7)	
AJCC clinical stage^b^			0.12
II	11 (68.8)	10 (41.7)	
III	5 (31.2)	14 (58.3)	
ER/PR status^b^			0.02
Positive	5 (31.2)	17 (70.8)	
Negative	11 (68.8)	7 (29.2)	
Lymphovascular invasion			0.21
Absent	15 (93.8)	18 (75.0)	
Present	1 (6.2)	6 (25.0)	
Nuclear grade			0.33
Grade 1/2	4 (25.0)	10 (41.7)	
Grade 3	12 (75.0)	14 (58.3)	
Neoadjuvant regimen			1.00
AC/TH	13 (81.3)	20 (83.3)	
Other^a^	3 (18.7)	4 (16.7)	
Operative approach^b^			0.09
BCS + XRT	8 (50.0)	5 (20.8)	
Mastectomy ± XRT	8 (50.0)	19 (79.2)	

^a^Other included carboplatin/taxotere/trastuzumab and taxotere/cyclophosphamide/trastuzumab. ^b^Included in multivariable logistic regression analysis to determine independent correlates to pCR. *pCR* pathologic complete response, *BMI* body mass index, *AJCC* American Joint Committee on Cancer, *ER* estrogen receptor, *PR* progesterone receptor, *AC/TH* Adriamycin/Cytoxan/Taxol/Herceptin, *BCS* breast-conserving surgery, *XRT* radiotherapy

Relevant clinicopathologic variables on univariate testing (i.e., ER/PR status, clinical stage, operative approach) and anti-HER2 responsivity (Th1 repertoire and cumulative response were highly co-linear) were entered into a multivariable logistic regression analysis. Anti-HER2 Th1 responsivity (odds ratio (OR) 8.82, 95 % CI 1.50, 51.83, *p* = 0.016) and ER/PR status (OR 4.71, 95 % CI 1.03, 21.58, *p* = 0.046) remained independently associated with pCR status.

Due to persistence of the association between ER/PR status and pCR on multivariable analysis, anti-HER2 Th1 responses were stratified by hormone receptor status. No significant differences in anti-HER2 Th1 responsivity (72.2 % vs 50.0 %, *p* = 0.23), repertoire (1.9 ± 0.4 vs 1.1 ± 0.4, *p* = 0.12), or cumulative response (80.6 ± 18.7 vs 64.8 ± 20.3, *p* = 0.57) were observed between ER/PR^neg^ (n = 22) and ER/PR^pos^ (n = 18) patients, respectively.

### Th1 is the predominant phenotype contributing to anti-HER2 IFN-γ^+^ CD4^+^ T cell deficit

**Fig. 4:**
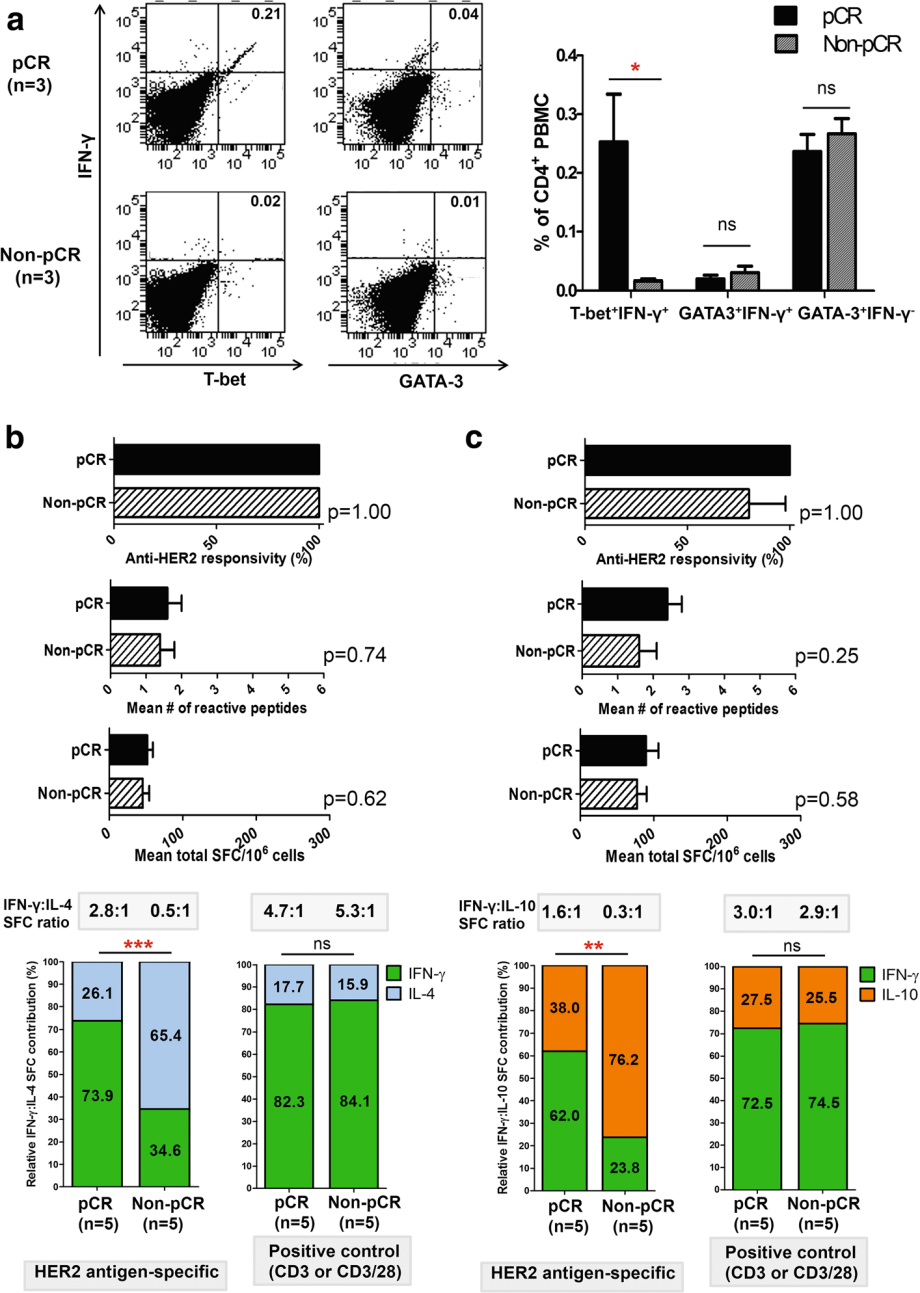
Anti-human epidermal growth factor receptor 2 (*anti-HER2*) CD4^+^ T-helper type-1 (Th1) is the dominant phenotype contributing to interferon-γ^+^ (*IFN-γ* ^*+*^) *CD4* ^*+*^ *T* cell deficit in patients without pathologic complete response (*non-pCR*). **a** Relative contributions of Th1 (*T-bet* ^*+*^ *IFN-γ* ^*+*^) versus Th2 (*GATA-3* ^*+*^ *IFN-γ* ^*+*^) phenotypes to HER2 peptide-specific IFN-γ^+^ cells in peripheral blood mononuclear cells (*PBMC*) from pCR and non-pCR patients. Representative stains within groups are shown after gating on CD4^+^ cells; results in adjoining histograms are expressed as mean proportions (%) ± standard error of the mean (SEM) as indicated. **b** Circulating *HER2-specific IL-4* production does not vary between pCR and non-pCR patients, when assessed by *responsivity*, *repertoire*, and *cumulative response*. Results expressed as proportion or mean ± SEM (*top panel*). Donor-matched cumulative IFN-γ and IL-4 production (spot-forming cells (*SFC*)/10^6^ cells) across six HER2 class II peptides compared in pCR and non-pCR patients. Relative *HER2-specific* IFN-γ:IL-4 proportions (% depicted in graph) was significantly higher in pCR (IFN-γ/(IFN-γ + IL-4) = 73.9 %:IL-4/(IFN-γ + IL-4) = 26.1 %) compared with non-pCR patients (34.6 %:65.4 %). *Absolute* IFN-γ:IL-4 production ratio changed from 2.8:1 (pCR) to 0.5:1 (non-pCR) (*bottom left panel*). No relative shifts in IFN-γ:IL-4 production were observed to *positive controls* (anti-CD3/CD28 or anti-CD3, respectively) (*bottom right panel*). **c** HER2-specific IL-10 production is similar between pCR and non-pCR patients across all Th1 metrics. Results are expressed as proportion or mean ± SEM (*top panel*). Relative *HER2-specific* IFN-γ:IL-10 production was significantly higher in pCR (62.0:38.0 %) compared with non-pCR (23.8:76.2 %) patients. *Absolute* IFN-γ:IL-10 production ratio changed from 1.6:1 (pCR) to 0.3:1 (non-pCR) (*bottom left panel*). No relative shifts in IFN-γ:IL-4 production were observed to *positive controls* (anti-CD3/CD28 or anti-CD3, respectively) (*bottom right panel*); *p <0.05, ** *p* <0.01, *** *p* <0.001

In order to ascertain the CD4^+^ T cell phenotype most contributory to the anti-HER2 IFN-γ^+^ immune deficit, HER2-stimulated PBMC were assessed for co-expression of T-bet (Th1 transcription factor [16]) or GATA-3 (Th2 transcription factor [17]), and intracellular IFN-γ by flow cytometry. Patients with pCR demonstrated a significantly greater proportion of HER2-specific CD4^+^T-bet^+^IFN-γ^+^ (0.25 ± 0.1 % vs 0.02 ± 0.01 %, *p* = 0.039), but not CD4^+^GATA-3^+^IFN-γ^+^ (0.02 ± 0.01 % vs 0.03 ± 0.01 %, *p* = 0.49) or CD4^+^GATA-3^+^IFN-γ^−^ (0.24 ± 0.03 % vs 0.27 ± 0.03 %, *p* = 0.52) PBMC, compared with patients with non-pCR (Fig. 4a).

To determine the functional contribution of Th2 and T_reg_ phenotypes, HER2-specific IL-4 and IL-10 production were examined via ELISPOT, respectively. While overall anti-HER2 IL-4^pos^ responsivity, repertoire, and cumulative response did not differ between pCR and non-pCR cohorts, donor-matched HER2-specific IFN-γ:IL-4 production ratios shifted from 2.8:1 (relative Th1-favoring phenotype) in pCR to 0.5:1 (relative Th2-favoring) in non-pCR patients (*p* <0.001; Fig. 4b). Similarly, overall anti-HER2 IL-10^pos^ immune metrics did not differ between pCR and non-pCR patients; however, relative anti-HER2 IFN-γ:IL-10 contributions shifted from 1.6:1 (Th1-favoring) in pCR to 0.3:1 (T_reg_-favoring) in non-pCR patients (*p* = 0.008; Fig. 4c).

### Th1 deficit in patients with non-pCR is unrelated to immune incompetence, host-level T cell anergy or immunosuppressive phenotypes

**Fig. 5:**
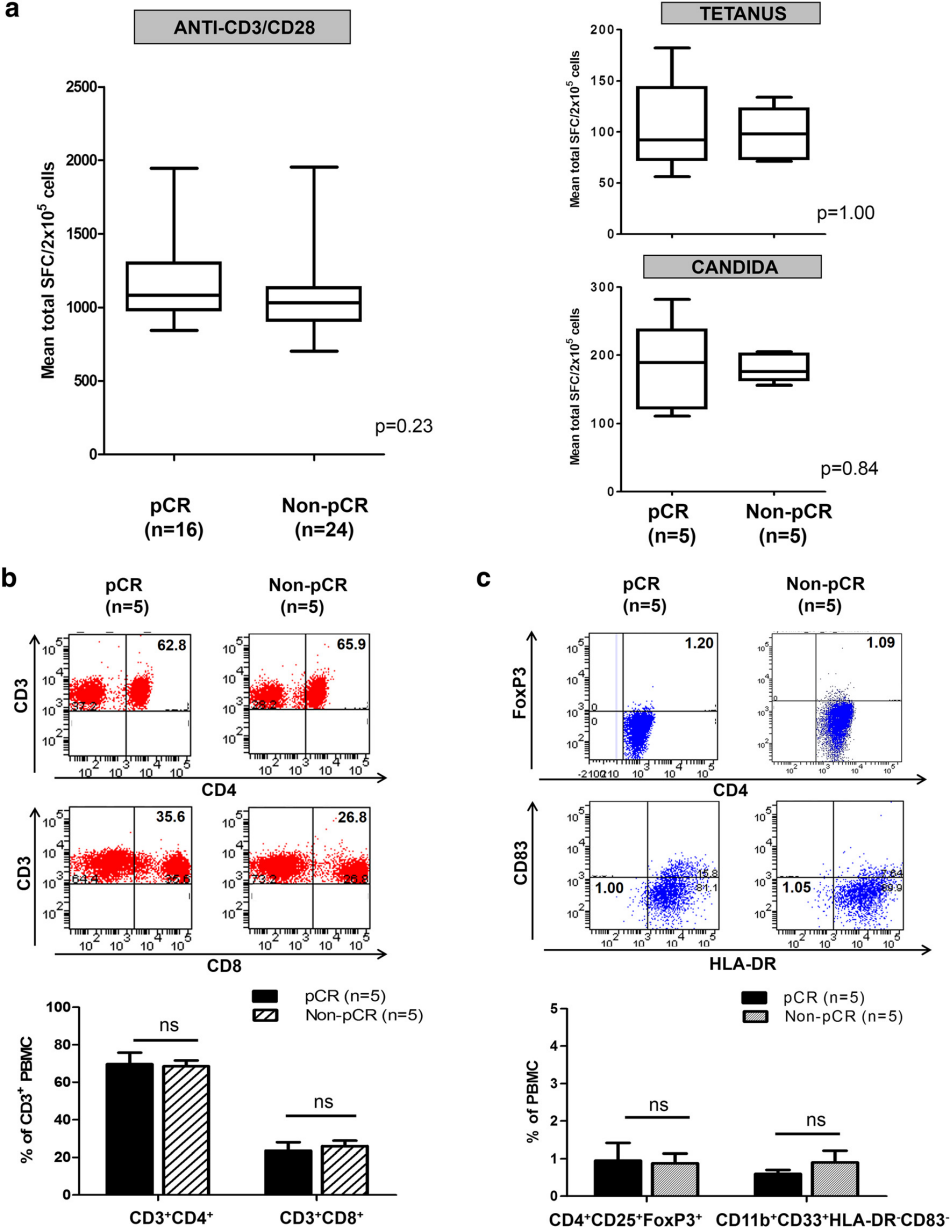
Anti-human epidermal growth factor receptor 2 (anti-HER2) T-helper type-1 (Th1) deficit in patients without pathologic complete response (*non-pC*R) is not attributable to lack of immune competence, host-level T cell anergy, or increase in immunosuppressive phenotypes. Peripheral blood mononuclear cells (*PBMC*) from pCR and non-pCR patients did not differ significantly in (**a**) immune competence – measured by IFN-γ production to anti-CD3/anti-CD28 stimulus or recall stimuli tetanus toxoid and *Candida albicans* – by enzyme-linked immunosorbent spot. Results presented as median ± IQR interferon-γ (IFN-γ) spot-forming cells (SFC)/2 × 10^5^ cells. **b**, **c** Relative proportions of CD3^+^CD4^+^ (**b**, *top*) or CD3^+^CD8^+^ T-cells (**b**, *bottom*), T_reg_ (CD4^+^CD25^+^FoxP3^+^) (**c**, *top*) or myeloid-derived suppressor cells (MDSC) (CD11b^+^CD33^+^HLA-DR^−^CD83^−^) (**c**, *bottom*) by flow cytometry. Representative stains within groups are shown; results in histograms are expressed as mean proportions (%) ± standard error of the mean as indicated

Immune competence in pCR and non-pCR subgroups was assessed by anti-CD3/anti-CD28-stimulated Th1 responses using IFN-γ ELISPOT. Mean anti-CD3/CD28 responses did not differ (1195 ± 87.4 vs 1085 ± 70.7 SFC/2 × 10^5^ cells, *p* = 0.23) between pCR and non-pCR cohorts. Furthermore, IFN-γ production following recall stimuli (tetanus toxoid (105 ± 20.9 vs 98 ± 11.6 SFC/2 × 10^5^), and *Candida albicans* (182 ± 29.8 vs 181 ± 9.0 SFC/2 × 10^5^)) were similar between pCR and non-pCR groups, respectively (Fig. 5a). Collectively, these data suggest that the anti-HER2 Th1 disparity is not attributable to host-level T cell anergy or impaired antigen-presenting capacity in patients with non-pCR PBMC.

On flow cytometry, the mean proportion of CD3^+^CD4^+^ (69.6 ± 6.2 % vs 68.5 ± 3.1 %, *p* = 0.87) and CD3^+^CD8^+^ (23.5 ± 4.5 % vs 25.9 ± 2.9 %, *p* = 0.67) cells did not differ between PBMCs from pCR and non-pCR patients, respectively (Fig. 5b). Proportions of B cells (CD19^+^) and natural killer (NK) cells (CD3^−^CD16^+^) were similar between groups (data not shown). Circulating immunosuppressive phenotypes were compared: mean proportions of CD4^+^CD25^+^FoxP3^+^ cells (T_reg_) (0.95 ± 0.5 % vs 0.88 ± 0.3 %, *p* = 0.89), and CD11b^+^CD33^+^HLA-DR^−^CD83^−^ cells (myeloid-derived suppressor cells (MDSC)) (0.6 ± 0.1 % vs 0.9 ± 0.3 %, *p* = 0.34) did not differ between pCR and non-pCR subgroups, respectively (Fig. 5c).

### Anti-HER2 Th1 deficit in patients with non-pCR can be corrected with HER2-targeted CD4^+^ Th1 immune interventions

We have previously demonstrated that intranodally injected HER2-pulsed DC1s elaborate abundant IL-12p70 and polarize naïve CD4^+^ T-cells to IFN-γ/TNF-α-producing anti-HER2 Th1 in vivo [12, 18]. When employed in HER2^pos^ ductal carcinoma in situ (DCIS) and patients with stage I HER2^pos^ IBC in phase I/II trials, autologous HER2-targeted DC1 vaccination resulted in durable anti-HER2 Th1 immunity; pCR rates approached 25 % with substantial loss of target antigen in the remainder of patients (unpublished data) [15, 19].

**Fig. 6:**
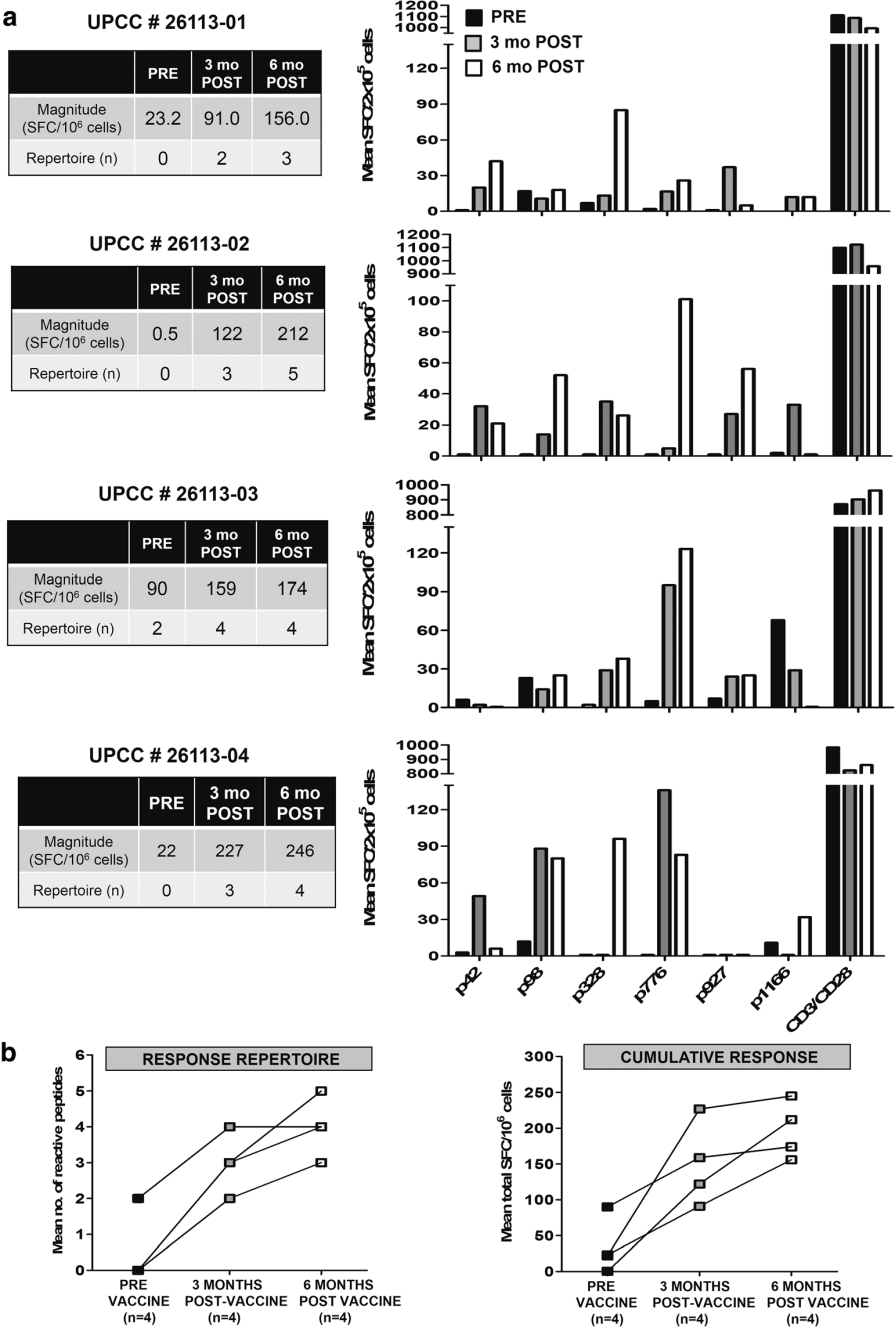
Depressed anti-human epidermal growth factor receptor 2 (anti-HER2) T-helper type-1 (Th1) immunity is restored following HER2-pulsed type 1-polarized dendritic cell (DC1) immunization. **a** Anti-HER2 CD4^+^ T cell immune reactivity profiles in four patients with non-pCR (*UPCC # 26113–01 to −04*) undergoing HER2-pulsed DC1 vaccination in the ongoing phase I trial NCT02061423 are demonstrated at three time points: pre-vaccination (*black*), 3 months post vaccination (*gray*), and 6 months post vaccination (*white*). For these time points, responses to individual HER2-derived class II peptides (*x-axis*; p42, p98, p328, p776, p927, p1166) are graphed, with anti-CD3/CD28 serving as positive control. Anti-HER2 Th1 repertoire and cumulative responses at these time points are listed in adjoining tables. **b** Anti-HER2 Th1 repertoire (*mean # of reactive peptides*) and cumulative response (*mean total SFC/10* ^*6*^ *cells*) increase progressively in patients with non-pCR at the 3-month and 6-month time points. *SFC* spot-forming cells

In order to determine the impact of HER2-Th1-targeted immune interventions in high-risk non-pCR patients, four non-pCR patients (*cohort G*; Fig. 1) were recruited to our phase I adjuvant HER2-pulsed DC1 vaccination trial (NCT02061423); demographic and clinicopathologic characteristics of enrolled patients are detailed in Table 3. Subjects received six weekly injections followed by three booster doses at three-month intervals. Vaccination-induced anti-HER2 Th1 responses were followed prospectively; Th1 reactivity in individual patients pre-vaccination and post-vaccination is illustrated in Fig. 6a. In vaccinated subjects, evaluable anti-HER2 Th1 responses measured 6 months post-vaccination (i.e., prior to the third booster) indicated significantly improved anti-HER2 Th1 repertoire (3.7 ± 0.5 post-vaccination vs 0.5 ± 0.5 pre-vaccination, *p* = 0.014) and cumulative response (192.3 ± 16.4 vs. 33.9 ± 19.4 SFC/10^6^, *p* = 0.014) compared with pre-vaccination levels (Fig. 6b). Vaccinations were well-tolerated, with only two cases of grade-1 toxicity observed.

**Table 3:** Demographic and clinicopathologic characteristics of patients recruited to the ongoing phase I trial of HER2-pulsed DC1 vaccination in HER2^pos^ IBC patients with residual disease following neoadjuvant trastuzumab and chemotherapy (NCT02061423)

Subject	Age, y	HLA	Race	Menopausal status	ER	PR	HER2	T + C regimen	Surgery	yp stage
26113-01	67	A2	White	Postmenopausal	+, 30 %	+, 5 %	3+, >90 %	AC/TH	MRM	T1b N2a
26113-02	35	A2	White	Premenopausal	+, 30 %	+, 4 %	2+, FISH +	AC/TH	MRM	T2 N2a
26113-03	45	-	White	Premenopausal	+, 84 %	-, 3 %	3+, >10 %	AC/TH	MRM	Tis N0
26113-04	55	-	White	Postmenopausal	−, 0 %	-, 0 %	3+, >10 %	TC	MRM	T1c N1mi

*HER2* human epidermal growth factor receptor 2, *DC1* type 1-polarized dendritic cell, *IBC* invasive breast cancer, *HLA* human leukocyte antigen (A2 yes/no), *ER* estrogen receptor, *PR* progesterone receptor, *T + C* trastuzumab and chemotherapy, *yp* post-neoadjuvant pathologic stage, *AC/TH* Adriamycin/Cyclophosphamide/Taxol/Herceptin, *MRM* modified radical mastectomy, *FISH* fluorescence in situ hybridization, *TC* Taxotere, Cyclophosphamide

## Discussion

In the present study, we identify a novel systemic immune correlate to pathologic response following neoadjuvant HER2-targeted therapy in patients with HER2^pos^ IBC. Although not globally improved in all patients treated with T + C, anti-HER2 CD4^+^ T-cell immunity is more robust in patients achieving pCR compared with their non-pCR counterparts despite controlling for relevant demographic and tumor-related confounders. HER2-specific Th1, but not Th2, CD4^+^ T-cells appear to be the dominant contributor to the circulating anti-HER2 IFN-γ^+^ immune disparity; this anti-HER2 Th1 deficit is not attributable to host-level T cell anergy, lack of immunocompetence, or preponderance of immunosuppressive phenotypes in non-pCR patients. Importantly, this anti-HER2 Th1 deficit is modifiable, and can be corrected with HER2-pulsed DC1 vaccinations. In high-risk non-pCR patients, strategies to boost anti-HER2 Th1 immunity may be of benefit.

Pathologic complete response following neoadjuvant administration of HER2-targeted therapies is a reliable surrogate for favorable long-term outcomes in HER2^pos^ BC [7, 8]; in fact, the Food and Drug Administration (FDA) supports pCR as a trial endpoint for drug approval [20]. Conversely, non-pCR portends a worse overall prognosis. Recent investigation has elucidated tumor cell-level mechanisms that account for suboptimal responses to HER2-targeted therapies, including overexpression of EGFR, cMYC, or ERBB3, and mutational loss of PTEN or activation of PI3K [21]. Beyond these factors, and the known association between ER negativity [8] – which is not readily modifiable – and pCR, there is a relative void in our understanding of host-level factors that impact response to HER2-directed therapies. In the current study, heightened circulating anti-HER2 CD4^+^ Th1 immune responses correlate strongly with pCR; conversely, the association of an anti-HER2 Th1 immune deficit with non-pCR warranted a search for therapeutic strategies that might correct this deficit. Fortunately, even in these heavily pre-treated patients, the Th1 deficit did not appear to be immunologically fixed and could be rectified with appropriate HER2-directed Th1 interventions. Thus, while a strategy such as withholding HER2-targeted therapies in patients with negatively prognostic tumor-level genetic alterations (e.g., PI3K mutations) is impractical [22], augmenting the depressed anti-HER2 Th1 immunity in non-pCR patients may be more feasible as an adjunct to existing HER2-targeted therapies to improve clinical outcomes.

CD4^+^ Th1 cells have emerged as critical components of antitumor immunity. Via expression of T-bet and IFN-γ, Th1 cells indirectly mediate antitumor effects by enhancing CD8^+^ cytotoxic T-lymphocyte and NK cell function [23]. In addition, via elaboration of IFN-γ and TNF-α, HER2-specific Th1 cells – in synergism with trastuzumab-mediated HER2 blockade – directly promote senescence and apoptosis, as well as HER2-specific CD8^+^ T cell targeting of HER2-overexpressing tumors in vitro [10, 24]. Indeed, the association between improved HER2_369–377_-specific *CD8*^*+*^ T-cell immune responses and tumor eradication in pCR patients may reflect the ready availability of CD4^+^ T cell help. Moreover, a recent genomic analysis from the NCCTG-N9831 trial demonstrated a strong association between increased relapse-free survival in adjuvant trastuzumab-treated patients and a signature of immune function genes, including IFN-γ and TNF-α [21]. In the present study, a relative decay in circulating anti-HER2 T-bet^+^IFN-γ^+^ (i.e., Th1), but not GATA-3^+^IFN-γ^+^ (i.e., Th2), phenotypes is associated with persistence of HER2^pos^ tumors following neoadjuvant T + C. Taken together, these data suggest that abrogation of immunologic, particularly anti-HER2 Th1 function, may represent a HER2^pos^ tumor-driven mechanism to evade immune surveillance during T + C treatment. Immune interventions aimed at restoring anti-HER2 Th1 function may be valuable in improving pathologic response following neoadjuvant T + C.

In parallel with these observations, growing evidence indicates that robust cellular immune responses in the tumor microenvironment are associated with improved outcomes in BC [25], particularly in HER2^pos^ subtypes [26]. Furthermore, an analysis from the GeparQuattro trial suggested that tumor-infiltrating lymphocyte (TIL) density correlates with pCR following neoadjuvant T + C; for every 10 % increase in TIL levels, a 16 % increase in pCR rates was observed [27]. The sizeable increase in circulating anti-HER2 Th1 populations in pCR patients in the present study may represent a systemic corollary to such immune-related changes in the tumor microenvironment, and lend further support to evidence that intact immune functionality, in addition to HER2-signaling inhibition, is critical in mediating antitumor effects following T + C treatment [28]. What is not immediately evident from our analysis, however, is whether the heightened anti-HER2 Th1 responses in pCR patients represent preservation of erstwhile immunity, or immune restoration following T + C treatment. If the latter is true, these data may further support immune restorative neoadjuvant interventions in order to improve pathologic response.

Other limitations merit discussion. First, given the retrospective and exploratory nature of the study design, the findings herein should be interpreted as hypothesis-generating and warrant large-scale validation. Second, despite minimal demographic/clinical variability between treatment-naïve T + C - treated HER2^pos^ - IBC cohorts, the global lack of improvement in anti-HER2 Th1 responses following T + C treatment must be interpreted with caution, since these data were derived from an unpaired comparison between independent patient samples. Finally, while encouraging, definitive conclusions regarding the immune restorative impact of HER2-directed DC1 vaccination in high-risk non-pCR patients, cannot be drawn until completion and final reporting of this ongoing trial.

The translational implications of these findings bear emphasis. As discussed, they may justify addition of HER2-targeted Th1 immune interventions to neoadjuvant T + C regimens and/or in the adjuvant setting for high-risk non-pCR subgroups. Moreover, in light of our recent demonstration that depressed anti-HER2 Th1 immunity correlates with subsequent recurrence in patients treated with adjuvant T + C [10], monitoring high-risk patients with non-pCR for real-time fluctuations in anti-HER2 Th1 immunity may complement existing radiographic surveillance, and identify critical opportunities for therapeutic intervention. Incorporation of anti-HER2 Th1 immune detection protocols in future clinical trial design, particularly those investigating neoadjuvant HER2-targeted therapies, appears justified.

## Conclusions

In summary, this is the first description, to our knowledge, of a critical association between anti-HER2 CD4^+^ Th1 immunity and pCR following neoadjuvant T + C in HER2^pos^ IBC patients. Although our data cannot confirm causality, the dramatic IFN-γ^+^ anti-HER2 Th1 deficit observed in non-pCR patients following neoadjuvant T + C raises the possibility that immune rescue with HER2-Th1 interventions may complement standard HER2-targeted strategies in improving outcomes in these high-risk patients. While correction of the anti-HER2 Th1 immune deficit has already been observed in non-pCR patients recruited to our HER2-DC1 vaccination trial, longitudinal follow up and larger-scale studies will establish if such immune manipulations ultimately mitigate recurrence in such patients.

## Additional file

Additional file 1: Figure S1. Representative interferon (IFN)-γ enzyme-linked immunosorbent spot (ELISPOT) assay results/calculation. IFN-γ ELISPOT results from a single representative pathological complete response (pCR) (*left panel*) and non-pCR (*right panel*) patients' peripheral blood mononuclear cells (PBMC) illustrate our method of calculating the T-helper type-1 (Th1) metrics utilized herein. PBMC are plated in triplicate, and spot-forming cells (*SFC*) following ex vivo human epidermal growth factor receptor (HER)2-derived class II peptide stimulation (indicated as *stimulus*, i.e., peptide 42–56, 98–114, 328–345, 776–790, 927–941, 1166–1180) are analyzed by an automated plate reader. Peptide-specific mean IFN-γ responses are determined after subtracting from *unstimulated* background (e.g., p42-56 SFC minus unstimulated SFC). In the adjoining histograms that quantify the ELISPOT assays directly above, corrected mean peptide-specific IFN-γ SFC are plotted. Response to an individual peptide is considered positive/reactive if >20 SFC/2 × 10^5^ cells (*red line*). Th1 responsivity indicates whether a particular donor demonstrated a positive/reactive response to any of the six tested peptides - in this example, both the pCR and non-pCR patients were responsive. Th1 repertoire represents the number of reactive peptides - in this example, pCR donor: 4; non-pCR donor: 1. Th1 cumulative response is determined by summing peptide-specific SFCs across all six peptides, and standardizing to 10^6^ cells - in this example, pCR donor: 197.5 SFC/10^6^; non-pCR donor: 29.1 SFC/10^6^. Mean IFN-γ SFC to anti-CD3/anti-CD28 stimulus serves as positive control.

## Abbreviations

BC: breast cancer
BMI: body mass index
DC: dendritic cell
DC1: type 1-polarized dendritic cell
DMEM: Dulbecco’s modified Eagle’s medium
ELISPOT: enzyme-linked immunosorbent spot
ER: estrogen receptor
FACS: fluorescence-activated cell sorting
FCS: fetal calf serum
FISH: fluorescence in situ hybridization
GM-CSF: granulocyte monocyte colony stimulating factor
HER2: human epidermal growth factor receptor 2
HER2^pos^: human epidermal growth factor receptor 2-positive
IBC: invasive breast cancer
iDC: immature dendritic cells
IFN: interferon
IHC: immunohistochemistry
IL: interleukin
LPS: lipopolysaccharide
MDSC: myeloid-derived suppressor cells
MHC: major histocompatability complex
NK: natural killer
NIH: National Institutes of Health
OR: odds ratio
PBMC: peripheral blood mononuclear cells
PBS: phosphate-buffered saline
pCR: pathologic complete response
PMA: phorbol-12-myristate 13-acetate
PR: progesterone receptor
PVDF: polyvinylidene fluoride
RT: room temperature
SFC: spot-forming cells
T + C: trastuzumab and chemotherapy
Th1: T-helper type-1
TNF-α: tumor necrosis factor- α
