# Supplementary figures and images for: Anti-HER2 CD4+ T-helper type 1 response is a novel immune correlate to pathologic response following neoadjuvant therapy in HER2-positive breast cancer
Source: Breast Cancer Res. 2015 May 23;17(1):71. doi: 10.1186/s13058-015-0584-1 (PMC4488128; doi:10.1186/s13058-015-0584-1)

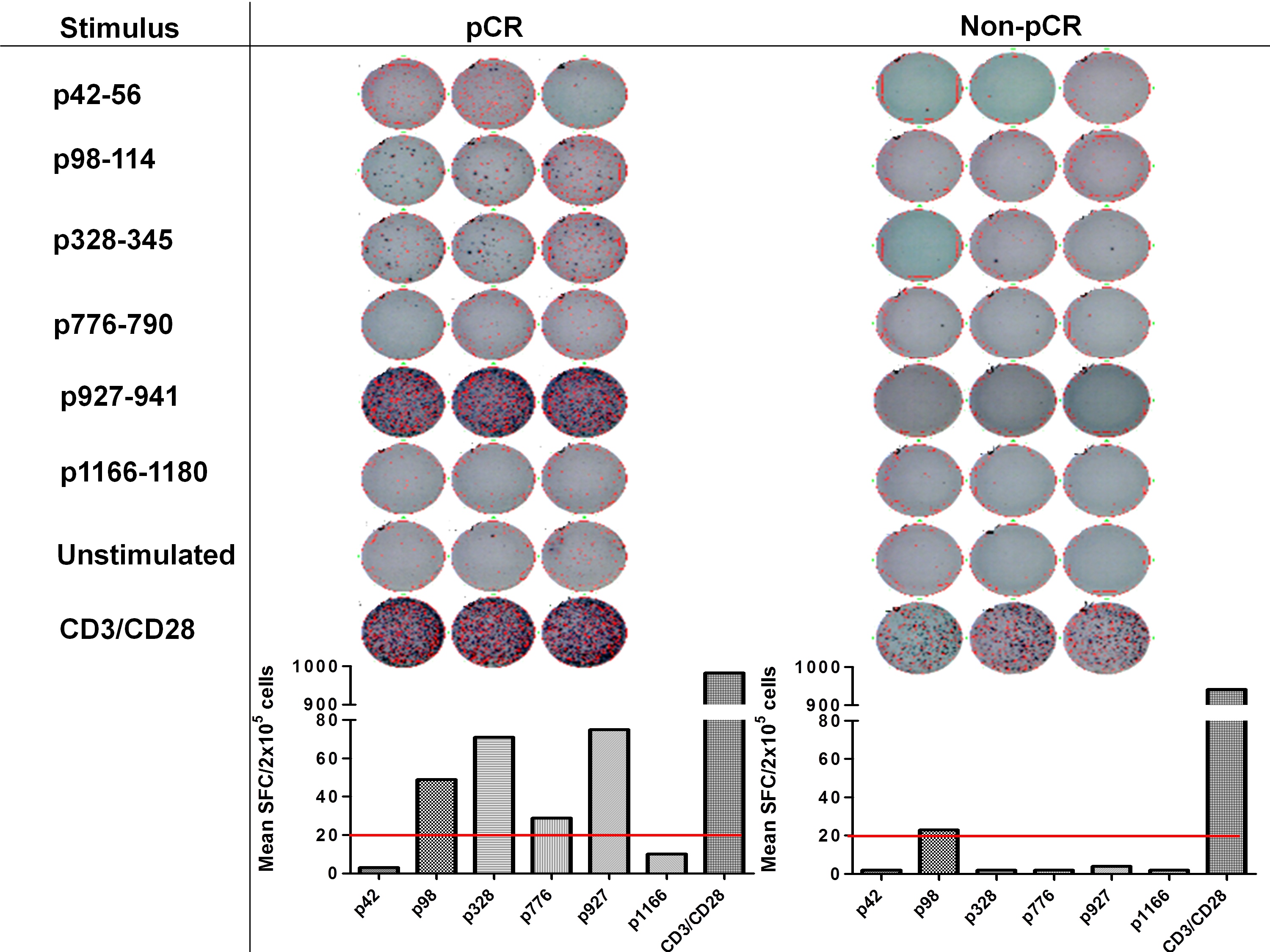

Supplement: Additional file 1: Figure S1. — Representative interferon (IFN)-γ enzyme-linked immunosorbent spot (ELISPOT) assay results/calculation. IFN-γ ELISPOT results from a single representative pathological complete response (pCR) (left panel) and non-pCR (right panel) patients' peripheral blood mononuclear cells (PBMC) illustrate our method of calculating the T-helper type-1 (Th1) metrics utilized herein. PBMC are plated in triplicate, and spot-forming cells (SFC) following ex vivo human epidermal growth factor receptor (HER)2-derived class II peptide stimulation (indicated as stimulus, i.e., peptide 42–56, 98–114, 328–345, 776–790, 927–941, 1166–1180) are analyzed by an automated plate reader. Peptide-specific mean IFN-γ responses are determined after subtracting from unstimulated background (e.g., p42-56 SFC minus unstimulated SFC). In the adjoining histograms that quantify the ELISPOT assays directly above, corrected mean peptide-specific IFN-γ SFC are plotted. Response to an individual peptide is considered positive/reactive if >20 SFC/2 × 105 cells (red line). Th1 responsivity indicates whether a particular donor demonstrated a positive/reactive response to any of the six tested peptides - in this example, both the pCR and non-pCR patients were responsive. Th1 repertoire represents the number of reactive peptides - in this example, pCR donor: 4; non-pCR donor: 1. Th1 cumulative response is determined by summing peptide-specific SFCs across all six peptides, and standardizing to 106 cells - in this example, pCR donor: 197.5 SFC/106; non-pCR donor: 29.1 SFC/106. Mean IFN-γ SFC to anti-CD3/anti-CD28 stimulus serves as positive control. [file 13058_2015_584_MOESM1_ESM.tif]
